# Circulating biomarkers in diagnosis and management of cardiac amyloidosis: a review for internist

**DOI:** 10.1007/s11739-022-02958-2

**Published:** 2022-03-24

**Authors:** Federico Perfetto, Mattia Zampieri, Carlo Fumagalli, Marco Allinovi, Francesco Cappelli

**Affiliations:** Regional Referral Center for Systemic Amyloidosis, Careggi University Hospital, Largo Brambilla 3, 50134 Florence, Italy

**Keywords:** Transthyretin, NT-proBNP, Troponin, Free light chains, AL cardiac amyloidosis, ATTR cardiac amyloidosis

## Abstract

Cardiac amyloidosis (CA) is due to extracellular myocardial deposition of misfolded proteins resulting in severe cardiac dysfunction and death. The precursors of amyloid fibrils, able of determining a relevant cardiac infiltration, are immunoglobulin-free light chains (AL amyloidosis) and transthyretin (TTR) (both wild and mutated types). The diagnosis of amyloidosis represents a challenge for the clinician given its rarity and its protean clinical presentation, thus an early diagnosis remains a cornerstone for the prognosis of these patients, also in light of the growing available treatments. There is great interest in identifying and applying biomarkers to help diagnose, inform prognosis, guide therapy, and serve as surrogate endpoints in these patients. In AL amyloidosis, biomarkers such as free light chains, natriuretic peptides and troponins are the most extensively studied and validated; they have proved useful in risk stratification, guiding treatment choice and monitoring hematological and organ response. A similar biomarker-based prognostic score is also proposed for ATTR amyloidosis, although studies are small and need to be validated for wild-type and mutant forms.

**Graphical abstract:**

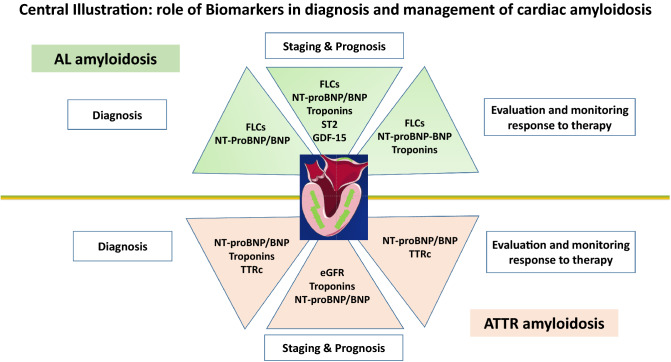

## Introduction

**Fig. 1:**
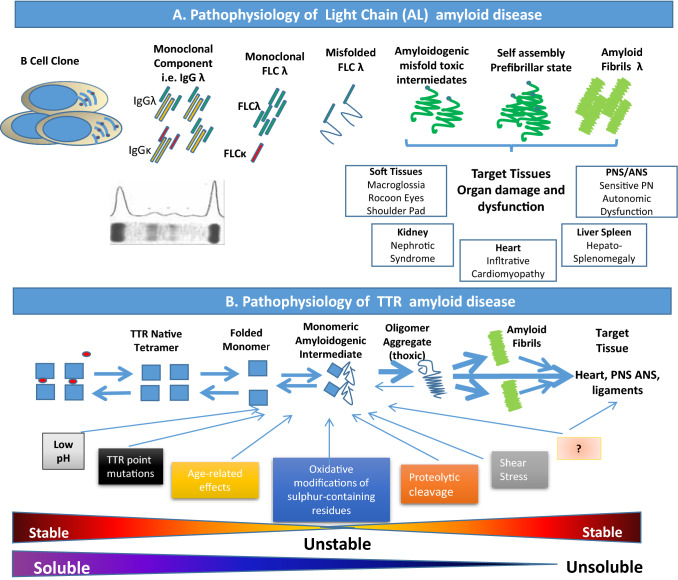
Pathophysiology of amyloid disease. **A** In AL amyloidosis, a small and indolent B cell clone overproduces a monoclonal light chain (*λ* in the majority of patients) with mutations in the variable region, which misfolds generating soluble amyloid oligomers and causing inappropriate aggregation into amyloid fibrils. The amyloid deposits, through the mass effect, induce a widespread disruption of tissue architecture and the oligomers, through their toxic effects, compromise cellular function and induce dysfunction in target organs. **B** Transthyretin is a homotetramer that circulates in the blood in equilibrium between the form bound to its ligands and the free form. Under physiological conditions, the TTR tetramer is relatively stable and the misfolding capacity is relatively low. Due to multiple mechanisms, largely unknown, the tetramer tends to separate into monomers that misfolds producing unstable, toxic intermediates, prone to aggregation with amyloid fibrils tissue deposition. During this amyloidogenic process, the native, soluble and stable TTR molecules, or FLC, are transformed into unstable and toxic intermediates and finally into very stable insoluble fibrils that are extremely resistant to degradation by tissue elimination systems. Central illustration: *FLC* free light chains, *AL* light chain amyloidosis, *ATTR* transthyretin amyloidosis, *NT-proBNP* N-terminal pro-brain natriuretic peptide, *BNP* brain natriuretic peptide, *ST2* soluble suppression of tumorigenicity 2, *GDF-15* growth differentiation factor-15, *eGFR* estimated glomerular filtration rate, *TTRc* circulating transthyretin, *ATTR* TTR amyloidosis. Green bars are myocardial amyloid fibrils deposit impairing heart function (Color figure online)

Amyloidosis is a disease due to the extracellular deposition of misfolded proteins in various tissues and organs, including the heart [1]. The two main types of amyloidosis capable of inducing clinically relevant cardiac involvement are light chain (AL) amyloidosis, in which amyloid fibrils originate from light chains (LC) of circulating monoclonal immunoglobulins, and transthyretin amyloidosis (ATTR). Destabilization of the tetrameric circulating TTR may either be due to age-related changes in wild-type disease (ATTRwt) or inherited mutations in the transthyretin gene (ATTRv) transmitted through an autosomal-dominant manner. In AL amyloidosis, a small and indolent B cell clone produces a light chain (*λ* chain in the majority of patients) with mutations in the variable region, inducing misfolded protein with high thermodynamic instability, generating soluble amyloid oligomers and causing inappropriate aggregation [2]. The amyloid deposits, through a mass effect, induce a widespread disruption of tissue architecture and the oligomers, through their toxic effects, compromise cellular function and induce organ dysfunction (Fig. 1A). Heart, kidney, peripheral nerves, liver and soft tissues are the main target organs of AL amyloid but rare and unusual localizations in many other tissues are equally possible [3]. In ATTR, circulating TTR, during the aging process, or in the context of a mutation, loses its quaternary structure altering its native form and favoring dissociation into oligomers and monomers which then precipitate in the tissues inducing organ damage (Fig. 1B). Several reports [4, 5] described an incidence of AL amyloidosis of about 1.2 (0.8–1.6) per 100,000 person-years with a trend that remains stable across the last decades. No data are available on incidence and prevalence of ATTR amyloidosis. It is believed that the prevalence of ATTRv is approximately 50,000 patients worldwide and that cardiac involvement is present in about 80% of cases, but these data may be underestimated [6]. The most common variant worldwide of TTRv with cardiac phenotype is the Val122Ile, which occurs in 3–4% of blacks American, with unknown phenotypic penetrance [7, 8]. The Italian Registry for ATTRv estimated a prevalence of 4.3/million, with regional differences [9]. ATTRwt is typically diagnosed at 70–75 years of age, with a striking male predominance [10, 11], and a prominent cardiac clinical phenotype. The last decade has been particularly relevant for TTR-CA thanks to the introduction in the diagnostic process of the scintigraphy with bone tracer and to its validation as a non-biopsy diagnostic tool by Gilmore et al. [12]. The easy and widespread access to bone scintigraphy together with a greater awareness of the disease and the imminent availability of disease modify treatments has led to a significant increase in the diagnoses of this type of amyloidosis suggesting that the real prevalence of ATTR is likely to be higher than what has been described so far [13, 14]. The diagnostic workup of CA includes the use of non-invasive cardiac imaging methods such as echocardiography, nuclear medicine, cardiac magnetic resonance as well as the use of specific genetic tests, frequently associated with histological demonstration of fibril amyloid deposits [15, 16]. Amyloidosis is a rare, complex and multi-organ disease that requires close interdisciplinary collaboration between various specialists to effectively diagnose, treat and support patients. The internist, together with the cardiologist, is an essential part of the multidisciplinary team that manages these patients throughout their clinical course. In particular internist, to whom patients with multi-organ dysfunction are most often addressed, must be aware of this disease and must be able to recognize it promptly especially in the early clinical manifestations, when organ damage is still amenable to treatment. Early diagnosis of amyloidosis and in particular of cardiac involvement is crucial meanly because overall survival is poor once overt cardiac involvement is present [1, 17–20]. The aim of treating amyloidosis is the greatest reduction of circulating amyloidogenic precursors and stop amyloid fibrils deposition, to halt or reverse organ damage. The choice of therapy, its duration, the evaluation of its effectiveness, the quantification of organ damage and its evolution during the course of therapy are all key moments for an optimal management of these complex patients. In this setting, the use of circulating biomarkers such as natriuretic peptides and high-sensitivity troponin, together with free light chains for AL amyloidosis, can be very useful in the management of these key steps and are used in clinical trials as indicators of response to treatment and as surrogate clinical endpoints (Central Illustration). Thanks to their multidisciplinary background, internists play a pivotal role in the management of this protean disease and are the only ones able to provide these patients with continuous and attentive support during treatment and follow-up. It is, therefore, imperative that internists have knowledge and awareness of the correct use of these biomarkers and that they are able to translate them into daily clinical practice for the correct classification and management of these complex patients. In this review, we aimed to summarize the current clinical use of circulating biomarkers for the assessment of diagnosis, prognosis, risk assessment and response to therapy in patients with CA and to mention the new areas of ongoing investigation in this field.

## Circulating biomarkers for diagnosis of amyloidosis

### AL amyloidosis

#### Biomarkers for diagnosis of AL amyloidosis

By definition, all patients with AL amyloidosis are characterized by the presence of free LCs (FLC) produced by a small B cell malignant clone, most often from bone marrow plasma cells [2]. In healthy subjects, plasma cells physiologically produce LCs in excess that do not bind to heavy chains to form a complete immunoglobulin (Ig) molecule, so that a small quantities of FLCs are present in the serum of normal subjects. In AL amyloidosis, the malignant B clone produces an excess of either intact Ig or FLCs of a single type (*κ* or *λ*) called monoclonal component (MC). In AL amyloidosis, amyloidogenic monoclonal FLCs are both the marker of the underlying clonal disease and the main pathogenic agent of tissue damage. Prior to the introduction of the FLC assay, the quantification of the amyloid protein precursor was inaccurate and derived from the amount of circulating intact serum monoclonal Ig, the amount of light chains in the urine and the number of plasma cells in the bone marrow [21]. Due to the small clone, in amyloidosis, the FLC production may be very small and may not reach concentrations high enough to be detected by serum protein electrophoresis (sPEP) or by serum and urinary immunofixation (sIFE and uIFE) [22]. Furthermore, unlike multiple myeloma, fewer than half of patients with AL have detectable circulating intact monoclonal Ig, and in many others, the concentration of monoclonal Ig is as low as accurate quantification is not possible. Since demonstrating the presence of a circulating MC is a cornerstone of the diagnosis of AL amyloidosis, the inability with these assays to detect a small MC can result in a misdiagnosis. The use of the serum FLC assay, combined with sIFE and uIFE, can detect the underlying monoclonal gammopathy, with a propensity to amyloid, in approximately 96–98% of patients with AL [23, 24]. Omitting any of these tests may result in the inability to diagnose underlying plasma cell dyscrasia and thus delay or miss the diagnosis of AL amyloidosis. If all tests are negative, AL amyloidosis is unlikely and further evaluation is not indicated unless the clinical index of suspicion is very high. The commercially available antibodies, used to measure FLCs, recognize, with high specificity, the LC epitopes that are exposed only when the LC are unbound. The Freelite (The Binding Site Ltd, Birmingham, UK) assay utilizes highly specific polyclonal sheep antibodies, while the N-Latex (Siemens Healthcare Diagnostics, Marburg, Germany) assay is based on monoclonal antibodies. Although the N-Latex and Freelite™ assays present several differences, they have similar diagnostic sensitivity and prognostic performance, but the between-assay agreement is modest [25]. In normal subjects, the kidney is the major site of FLCs’ catabolism with a serum half-life depending on their molecular weight. Monomeric k FLCs are cleared in 2–4 h, while dimeric *λ* FLCs in 3–6 h. Thus, in a patient with advanced kidney disease, the decline in glomerular filtration rate results in increased serum FLC concentrations. Due to the different kidney catabolism on *κ* and *λ* FLCs, the reference range for FLC *κ*/*λ* at Freelite assay has proven to be inappropriate to reveal an underlying amyloidogenic FLC, especially in patient with *λ* clones. A wider reference range, for FLCs *κ*/*λ* ratio with Freelite, has been recommended in patients with advanced kidney disease (0.37–3.1 instead of 0.26–1.65) [26].

#### Biomarkers for assessment of cardiac involvement in AL amyloidosis

Determination of cardiac involvement in systemic AL amyloidosis is of vital importance and is crucial for staging, determining prognosis and tailoring treatment. NT-proBNP is a highly sensitive, although not specific, marker of cardiac involvement in patients with extracardiac biopsy-proven AL amyloidosis. NT-proBNP values below the 97.5 percentile for normal subjects virtually excludes significant AL cardiac involvement and may obviate the use of imaging tests such as echocardiography or MRI in patients with low clinical suspicion of CA [27]. Increased NT-proBNP levels have been observed in AL amyloidosis with preclinical cardiac involvement, even in patients with apparently normal echocardiographic findings [28]. This feature seems related to a direct toxic effect of LCs on myocardial cells, that is independent of infiltrative damage. Amyloidogenic LCs from patients with cardiac AL amyloidosis may induce mitogen-activated protein kinase (MAPK) p38 signaling, which, in addition to mediating cardiomyocyte dysfunction and cell death, can also induce BNP transcription, supporting a possible link between the cardio toxic effects of LCs and increased natriuretic peptides release [29, 30]. Consequently, NT-proBNP could be considered a useful screening tool for detecting an underlying preclinical CA in some at-risk populations such as patients with MGUS and an abnormal FLC ratio [31]. Since most patients with AL amyloidosis at onset show high levels of NT-proBNP and/or albuminuria, evaluation of these two parameters in patients with MGUS and abnormal FLC ratio at each follow-up visit, may lead to a preclinical diagnosis of AL amyloidosis, with significant benefits in terms of treatment efficacy and overall survival [32]. The amyloidosis consensus statement defines cardiac involvement, in the presence of extracardiac biopsy-proven systemic amyloidosis, as the presence of a mean left ventricular (LV) end-diastolic wall thickness ≥ 12 mm on echocardiography in the absence of other possible causes, or an NT-proBNP > 332 pg/mL in the absence of atrial fibrillation or renal failure. In a pivotal study on patients with AL amyloidosis [33], Palladini et al. demonstrated that NT-proBNP retained a good diagnostic accuracy for cardiac involvement even in case of reduced glomerular filtration rate (GFR), using a higher NT-proBNP cutoffs (332 pg/mL, 543 pg/mL and 2642 pg/mL for patients with GFR ≥ 60, 60–15 and < 15 mL/min/1.73 m^2^, respectively) [34]. As BNP is less cleared by kidney, higher diagnostic cutoffs have been suggested only for patients with GFR < 15 mL/min/1.73 m^2^ (221 pg/mL instead of 73–78 pg/mL) [34].

### ATTR amyloidosis

#### Biomarkers for assessment of cardiac involvement in ATTR amyloidosis

The diagnostic role of circulating biomarkers and, in particular, of natriuretic peptides and troponins in diagnosis of ATTR CA has not been evaluated so far. In general, NT-proBNP levels are significantly lower in ATTR-CA than in AL-CA amyloidosis and in ATTRv-CA than in ATTRwt, despite similar LV mass and renal function [35]. This discrepancy could be due to a lower cytotoxicity of TTR amyloid fibrils and a slower amyloid deposition process in ATTR [35]. Cardiac biomarkers evaluation is also recommended in pre-symptomatic TTRv carriers with predominant cardiac phenotype mutation, starting 10 years before the predicted age of onset of symptomatic disease. In case of abnormal values, even in the absence of symptoms suggestive of cardiac involvement, it is recommended to carry out a series of diagnostic investigations, first, a bone tracer scintigraphy to reach a definitive and early diagnosis [36].

### Biomarkers for staging and prognosis

#### AL amyloidosis

The prognosis of patients with AL amyloidosis is largely based on the presence and extent of cardiac dysfunction. Several studies have revealed that both NT-proBNP and troponins have prognostic predicting value in AL patients. In 2004, Dispenzieri et al. [37] proposed and validated a prognostic risk staging based on NT-proBNP and cardiac troponin. They identified, in newly diagnosed AL patients, three stages associated to different incremental mortality risks. For this staging system, the threshold values for the NT-proBNP were 332 pg/mL, and 0.035 mg/L for cTnT (or 0.1 mg/L for cTn I) with stage I characterized by both biomarkers below the threshold, stage II with one biomarker above the threshold and stage III with both the biomarkers above the thresholds. Stage I, II and III were associated with significantly reduced median survivals of 102, 23 and 9 months, respectively. Wechalekar et al. [38], analyzing the outcomes response to chemotherapy in a large cohort of patients classified as stage III, identified an additionally ultra-high risk population characterized by NT-proBNP concentrations > 8500 pg/mL and troponin *T* > 0.035 mg/L (Table 2) associated with a systolic blood pressure lower than 100 mmHg. In this group of patients, indicated as stage IIIB, the median of OS (Overall Survival) was only 3 months. Indeed, Mayo stage IIIB patients have a very dismal outcome characterized by early deaths, due to severe and irreversible cardiac dysfunction. This staging system has been accepted for prognosis in clinical practice and indicated as Mayo2004/European. Accurate identification of such patients is important because treatment in this group is particularly challenging. Based on the demonstration that difference > 180 mg/L between involved and uninvolved FLCs, (dFLC) is an independent prognostic factor in AL amyloidosis, in 2012, the Mayo2004 staging system was revised and expanded including the clonal dFLCs [39]. This new Mayo2012 staging system sets different cutoff thresholds values for NT-proBNP > 1,800 pg/mL and for cTnT > 0.025 ng/mL (Table 2). This brought stages from I to IV with a median overall survival of 94.1, 40.3, 14, and 5.8 months, respectively (*p* < 0.001). The Mayo2012 system has been also validated using high-sensitivity cTnT (Roche Diagnostics by ELECSYS 2010 automated analyzer) with a cutoff > mg/L. The 99th percentile upper reference limit of this assay was greater than 14 ng/L for hs-cTnT with a limit of detection of 5 ng/L and a 10% coefficient of variation value of 13 ng/L [40]. The predicting value of all these three staging systems was confirmed by Dittirch et al. [41] even in patients with renal failure (50 mL/min/1.73 m^2^) and atrial fibrillation, two conditions commonly affecting cardiac biomarkers serum levels. A prognostic staging system was also determined using BNP (instead NT-proBNP) threshold values of 81 pg/mL and maintaining the same cutoff for TnI (< 0.1 mg/L) as in Mayo 2004 [42] (Table 1). Furthermore, Abdallah et al. [43] have recently demonstrated in more than 1,000 patients with AL amyloidosis that all the current staging systems retain prognostic value when used for re-stratify prognosis at 3 and 6 months from first-line treatment initiation. They have also showed that migration to a worse disease stage predicts decreased survival, whereas improvement in disease stage predicts longer survival in the subset of patients with advanced-stage at diagnosis Autologous stem cell transplantation (ASCT) is a well-recognized treatment with excellent outcomes in selected patients with AL amyloidosis. Although the eligibility criteria for ASCT varied across centers, there is a consensus to exclude patients aged > 70 years, significant involvement of more than two organs, including heart, significantly decreased renal function and low systolic blood pressure [44–46]. Concerning cardiac biomarkers, several studies [45, 46] suggest that patients with serum troponin T level above 0.06 ng/mL and/or NT-proBNP levels above 5000 pg/mL should be excluded from ASCT, due to higher transplant-related mortality rates.

**Table 1:** Staging and prognostic systems for AL amyloidosis basing on cardiac biomarkers and FLC

Staging system	Markers and thresholds	Stage	Outcome Median survival per stage (months)
Mayo Staging System (Mayo 2004)	NT-proBNP: 332 pg/mL cTnT*: 0.035 mg/L or cTnI: 0.1 mg/L	I No markers > cutoff II One marker > cutoff III. Both markers > cutoff	27 months 11 months 4 months
Mayo2004/European	Mayo2004 stage III is divided in 2 groups according to: NT-proBNP: 8500 pg/mL	III A < NT-proBNP 8500 pg/mL III B > NT-proBNP 8500 pg/mL	17 months 5 months
Mayo Staging System (Mayo2012)	dFLC: 180 mg/L NT-proBNP: 1800 pg/mL cTnT**: 0.025 mg/L	I 0 markers above the cutoff II 1 markers above the cutoff III 2 markers above the cutoff IV 3 markers above the cutoff	94 months 40 months 14 months 6 months
Boston University score (BNP based)	BNP: 81 pg/mL cTnI: 0.1 mg/L	I No markers > cutoff II One marker > cutoff IIIA Both markers cutoff BNP < 700 ng/L IIIB Both markers cutoff BNP > 700 ng/L	Not reached 9,4 years 4.3 years 1.0 years

*NT-proBNP* N-terminal pro-brain natriuretic peptide, *BNP* brain natriuretic peptide, cTnT* cardiac troponin T (sensitive second-generation assay, Roche Diagnostics, Indianapolis, IN), this assay has a limit of detection of less than 0.01 mg/L and coefficients of variability of 10% at 0.035 mg/L and 20% at 0.015 mg/L [35], cTnT** cardiac troponin T (sensitive fourth-generation assay, Roche Diagnostics, Indianapolis, IN), this assay has a limit of detection of less than 0.01 mg/L[36], *cTnI* cardiac troponin I (Dade-Behring, Newark, DE), this assay has a limit of detection of 0.03 mg/L and a coefficient of variability of 10% at the upper limit of the normal range of 0.06 mg/L [35], *dFLC* difference between involved and uninvolved free light chain

### ATTR amyloidosis

#### Biomarkers and risk stratification of ATTR CA

Prognostic stratification in ATTR amyloidosis remains challenging, due to the frequently delayed diagnosis, different progression in cardiac infiltration due to the specific amyloid precursor (wild-type or mutated TTR), to the intrinsic mutation phenotype, whit its geographical distribution, to the endemic/non-endemic aggregation, gender and parental heritability [47–49]. However, the presence and the extent of cardiac involvement are by far the main prognostic factors, regardless of the specific amyloid precursor. The recent availability of effective therapeutic approaches for ATTR generated the need for effective tools to assess patient prognosis, helping clinicians in daily practice and researchers in further studies developing. Biomarker-based prognostic staging systems have been developed for ATTR-CA, following the model adopted for AL amyloidosis. Two different staging systems have been proposed: in 2016, Grogan et al. [50] demonstrated that in a cohort of wtATTR-CA patients, NT-proBNP (cutoff 3000 pg/mL) and hsTnT (cutoff 0.05 ng/mL) plasma values were able to stratify the risk for death similarly as previously observed in AL patients. Gillmore et al. in 2017 demonstrated the prognostic performance of combining NT-proBNP (cutoff 3000 pg/mL) and estimated glomerular filtration rate (eGFR cutoff 45 mL/min/1.73 m^2^) in both wt- and hATTR-CA patients [51] (Table 2). The comparison of the two staging systems in a population of 172 ATTR CA patients (133 with ATTRwt and 42 with ATTRv-CA), showed that only 70% of patients were classified by the two different staging systems in the same stage with a kappa value of 0.541 [52]. Most of the discordances were observed for stages 2 and 3. The Gillmore staging allows for better stratification of patients in three subgroups with significantly different survival and risk for all-cause mortality. The same holds true for the Hazard Ratio analysis, while no statistically significant difference was detected between stages 1 and 2 in the overall population, according to the modified Grogan staging system. The combination of NT-proBNP and eGFR seems to lead to a better discrimination of patient prognosis than the combination of NT-proBNP and troponin, possibly as an expression of a type 2 cardiorenal syndrome, frequently observed in ATTR CA patients; therefore, it could be the chosen staging system in clinical practice. This stage has been indicated as National Amyloidosis Centre (NAC) transthyretin amyloidosis stage. Recently, Cheng et al. [53] reported that diuretic dose and NYHA functional class are independent predictors of mortality in ATTR-CA in addition to Grogan and Gilmore risk scores. Testing these risk scores in 309 patients with ATTR-CA (66.0% with ATTRwt and 34.0% with ATTRv), they showed that adding diuretic dose as categories (0 mg/kg; > 0 to 0.5 mg/kg; > 0.5 to 1 mg/kg; and > 1 to 2 mg/kg) improves the area under the curve of the Grogan risk score from 0.693 to 0.767 and the Gillmore risk score from 0.711 to 0.787, preserving calibration in both cases. Adding NYHA functional class, the area under the curve further improved to 0.798 and 0.816, respectively. Given that diuretic dose and NYHA functional class are easily obtainable information, they suggested that these variables should be considered in the clinical setting to better stratify the patients risk.

**Table 2:** Staging and prognostic systems for ATTR cardiac amyloidosis

Staging system	Markers and thresholds	Stage	Outcome Median survival (months) per stage
Grogan 2016 ATTRwt 360 patients	NT-proBNP: 3000 pg/mL cTnT:0.05 mg/L	I No markers > cutoff II One marker > cutoff III. Both markers > cutoff	66 months 40 months 20 months
Gillmore 2017 553 ATTRwt patients 316 ATTRv patients	NT-proBNP: 3000 pg/mL eGFR: 45 mL/min	I No markers > cutoff II One marker > cutoff III. Both markers > cutoff	62 months 47 months 24 months

*ATTRwt* transthyretin wild-type-related amyloidosis, *ATTRv* heredo-familial transthyretin-related amyloidosis, *NT-proBNP* N-Terminal pro-brain natriuretic peptide, *cTnT* cardiac troponin T (sensitive second-generation assay, Roche Diagnostics, Indianapolis, Indiana; for limit of detection and coefficients of variability see Table 2); *eGFR* estimated glomerular filtration rate

## Circulating biomarkers for assessment of response to therapy

### AL amyloidosis

#### Assessment of hematological response to therapy

Unlike multiple myeloma, organ failure and mortality in AL are directly due to the harmful effects of misfolded monoclonal LCs rather than the proliferation of monoclonal bone marrow plasma cells. Therefore, the immediate goal of AL amyloidosis treatment is to significantly and rapidly reduce the production of amyloidogenic FLCs to reduce organ damage. The greater (and faster) reduction in the FLC, the greater the possibility of improving the function of the affected organs and consequently survival. The possibility of quantifying FLCs represented a cornerstone in the management of this disease and allowed to evaluate and monitor the therapeutic response after chemotherapy. In 2012, in a large population of patients with AL amyloidosis, Palladini et al., using the Freelite assay, [54] identified the prognostic relevance of different criteria for predicting the hematologic response to treatment. They defined four levels of hematologic response categories: complete response (CR; negative serum and urine IFX and normal FLC ratio), very good partial response (VGPR; difference between involved and uninvolved FLCs [dFLC] < 40 mg/L), partial response (PR; dFLC decrease > 50%), and no response (NR). Changes in FLCs strongly predicted survival as early after few course of treatment (Table 3A). In AL amyloidosis, the quickly and profound reductions of the amyloid LCs burden (such as in VGPR or CR) are closely correlated with survival and are associated with the greatest chance of functional improvement of infiltrated organ. Thus, a VGPR should be the minimal goal of therapy in these patients. However, a high percentage of patients with AL are still diagnosed with too advanced cardiac dysfunction, which may not be improved even with an effective chemotherapy. Due to assay inaccuracy, patients with a dFLC less than the threshold of 50 mg/L are not evaluable for hematological response and are excluded from clinical trials. However, these patients with low FLC levels at baseline are not uncommon in AL amyloidosis, ranging from 13 to 19% of all patients [55, 56]. This population shows a high rate of renal involvement, but low rate of liver and cardiac involvement, which translate in a better clinical outcome [57]. In these patients, normalization of iFLC levels or decrease in dFLC < 10 mg/L (baseline at least 20 mg/L) were predictive of better overall survival and may be used for detecting hematological response [57]. Recently, the 2012 response criteria were updated by International Society of Amyloidosis (ISA) that propose that the previous response criteria be expanded to define complete hematological response as the absence of amyloidogenic light chains (either free or part of a complete immunoglobulin) described as negative serum and urine immunofixation and either a FLC ratio within the reference range or an abnormal FLC ratio as long as the uninvolved-FLC concentration is greater than involved-FLC concentration [58]. The meaning of this clarification is that an abnormal FLC ratio does not preclude the achievement of CR when the concentration of uninvolved, non-amyloidogenic, FLC is greater than that of the involved, amyloidogenic, FLC. This is particularly relevant today when highly effective anti-plasma cell therapies are available that can cause profound reductions in both-involved-FLC and uninvolved-FLC, possibly resulting in an inverted FLC ratio favoring non-amyloidogenic FLC. It is important to remind that current response criteria are validated using the Freelite assay and cannot be automatically applied to the N latex FLC method. In patients who achieved a VGPR or better response, a stringent clinical and biochemical surveillance should be started including a bimonthly assessment of serum FLC levels. In most patients, organ damage progression is frequently preceded by FLC increases, which can be subtle and should not be underestimated. However, there is no consensus on when and at which dFLC threshold, suggestive of relapse, the treatment should be started.

**Table 3:** Consensus criteria for organ and hematological response in AL amyloidosis

(A) Hematological response (HR)	
Type of response	Criteria (baseline dFLC needs to be ≥ 50 mg/L)
Complete response (CR) 2021 ISA criteria	Both criteria must be met: 1. absence of amyloidogenic light chains (either free and/or as part of a complete immunoglobulin) defined by negative immunofixation electrophoresis of both serum and urine 2. either a FLC ratio within the reference range or the uninvolved-FLC concentration is greater than involved-FLC concentration with or without an abnormal FLC ratio
Very good partial response (VGPR)	dFLC < 40 mg/L
Partial response (PR)	dFLC decrease ≥ 50%
No response (NR)	Less than PR

(B) Organ response (OR)	
Organ	Criteria
Heart (NT-proBNP based)	Reduction of NT-proBNP of 30% and 300 pg/mL over the starting value Baseline NT-proBNP has to be ≥ 650 pg/mL to be measurable
Heart (BNP based)	Reduction of BNP of 30% and 50 ng/L over the starting value Baseline BNP has to be ≥ 150 pg/mL to be measurable
Kidney	A 30% reduction in 24-h urine protein excretion or a drop of proteinuria below 0.5 g per 24 h in the absence of progressive renal insufficiency (defined as a decrease in eGFR to 25% over baseline)
Liver	A greater than 30% reduction in hepatomegaly on physical exam or a 50% decrease of an elevated alkaline phosphatase level

(C) Combined hematological (HR) and organ response (OR) staging system CHOR	
	Criteria
CHOR model	Score for HR: CR = 0; VGPR = 1; PR = 2; NR = 3 Score for OR: Response in all organs involved (AOR) = 0; Response in at least 1 but not all the organs involved (MOR) = 1; No organ response (NOR) = 2
Patients are classified in 2 CHOR groups according to a score based on the HR and OR criteria	CHOR 1 (score 0–3) CHOR 2 (score 4–5)

*dFLC* difference between involved and uninvolved free light chain, *NT*-*proBNP* N-terminal pro-natriuretic peptide type B, *BNP* natriuretic peptide type B

#### Assessment of organ damage response to therapy

In 2012, ISA validated the cardiac, renal and liver criteria for organ response as reported in Table 3B. Criteria for cardiac response is based exclusively on circulating NT-proBNP: a ≥ 30% and ≥ 300 pg/mL reduction in NT-proBNP starting from a baseline NT-proBNP ≥ 650 pg/mL has been defined as appropriate cardiac response, while a ≥ 300 pg/mL and ≥ 30% increase in NT-proBNP identified cardiac progression [54]. Cardiac response criteria to treatment and related survival rate were also validated using the BNP [59]. Since then, several studies have demonstrated that such biomarker-based cardiac response predicts survival regardless of the treatment regimen [60–64]. The NT-proBNP and BNP variation at 6 months from treatment (both ASCT or chemotherapy) predicts disease progression and are associated with better survival expectations [54, 59]. Data on troponin and its usefulness to monitor cardiac response to chemotherapy in AL are less well established. Palladini et al. in 2010 [65] demonstrated that > 75% increase in hs-cTnT from baseline values after chemotherapy, is an independent negative prognostic factor in AL amyloidosis. In AL amyloidosis, markers of hematological and other organs response should be monitored in parallel and frequently during chemotherapy, knowing that hematological response rarely goes hand to hand to organ response that usually may delay by several months [66]. Particular attention must be paid to the interpretation of the cardiac response in patients treated with immunomodulatory drugs (Thalidomide, Lenalidomide and Pomalidomide) due to their direct cardio toxic effect or fluid retention which directly increase NT-proBNP levels [67]. After ASCT, the clinical and biomarkers response should be frequently assessed, at least every 3 months and every 1 to 2 months after non-transplantation therapies, to allow a rapid switch to rescue therapy in patients who do not achieve an appropriate response. Recently, Sidana and Milani [68], respectively, validated, in two large independent cohorts of patients with AL amyloidosis, a model for early assessment of treatment benefit at 6 months, integrating both hematologic (HR) and organ response (OR) assessment (Table 3C). In this multi-marker strategy (CHOR model), they assigned four different scores to HR: 0 = complete response (CR), 1 = very good partial response (VGPR), 2 = partial response (PR), 3 = no response (NR) or progression; while OR was classified as follow: 0 = all organ (heart, kidney, liver) response (AOR), 1 = mixed organ response (MOR), described as response in at least one organ and 2 = no organ response (NOR). A composite HR/OR (CHOR) model was developed using incremental scoring from 0 to 5 based on hazard ratio, starting from score 0 (complete OR and HR) to score 5 (OR = 2 and HR = 3). Patients were then divided into two groups: CHOR group 1 (scores 0–3) and CHOR group 2 (scores 4–5). In both the two large independent cohorts, patients CHOR group 1 showed significant better OS (*p* 0.001) than patients CHOR group 2, median OS in group 1 and group 2 were, respectively, not reached vs 34 months, *p* < 0.001 in the Mayo cohort and 87 vs 23 months, *p *< 0.001 in the Pavia cohort. With this model, overall survival in both cohorts showed greater predictive power than hematological response or organ response assessed separately.

### ATTR amyloidosis

In the last years, the treatment of ATTR-CA is shifting from standard heart failure therapies and organ transplant to disease-modifying treatments [69]. Currently, there are two key approaches to disease-modifying therapy in ATTR: stabilizing the TTR tetramer thus reducing its amyloidogenicity and/or suppression of TTR production with gene silencing therapies. RNA silencing therapies include Patisiran, a ribonucleic acid interference agent, and Inotersen, an antisense oligonucleotide inhibitor [70]. Although many of these treatments are currently reserved for ATTRv patients with neurological phenotype, there is a need to evaluate the potential role of biomarkers in the assessment of cardiac response to therapy and as prognostic markers even in ATTR patients with cardiac involvement. However, the criteria defined to assess cardiac response to therapy in ATTR have not been validated so far [70]. Serum TTR is secreted by the liver and it functions as secondary carrier of thyroxine and binding partner retinol-binding protein-4 (RBP4). In ATTR, the misfolding of TTR initially involves a dissociation of the tetramer into a monomer and the formation of partially misfolded monomeric intermediates which are amyloidogenic and which subsequently self-assemble into amyloid fibrils via soluble aggregation. Mutations in the TTR gene capable of reducing tetramer stability are associated with decreased circulating levels likely due to increased tissue deposition of TTR derived fibrils [71, 72], while in subjects with Thr119Met, a TTR super-stabilizing and protective mutations, the levels are increased [73]. Notably, the treatment with AG-10, a selective TTR stabilizer which acts by mimicking the stabilizing effects of the Thr119Met variant, increases the TTR serum levels both in ATTRv and ATTR wt suggesting that an increase of TTR plasma level could be considered as a surrogate end point of effective stabilization [74]. On the other hand, gene silencers have the opposite effect on circulating TTR levels, with the rationale of a TTR amyloid substrate reduction. In the Apollo study, patients receiving Patisiran had a persistent 81% reduction of TTR circulating levels with clinical improvement of neuropathy and quality of life [75], indicative that, in this case, the rate of TTR reduction may be used as a useful biomarker candidate for clinical response to Patisiran. Similar results, in term of reduction of TTR levels, were reported in patients receiving Inotersen in the NEURO TTR study [76]. Recently, Law et al. [77] reported that change in NT-proBNP concentration, during the first year of follow-up, is a powerful independent predictor of mortality in a large cohort of patients with untreated wtATTR-CM. The authors emphasize that the prognostic relevance of the NT-proBNP changing was independent of NAC ATTR stage, troponin T, age, NYHA class, 6MWT distance, presence of atrial fibrillation and interventricular septal thickness at echocardiography.

### Novel biomarkers for CA

Preliminary studies suggest that growth differentiation factor-15 (GDF-15) [78], soluble suppression of tumorigenicity 2 (ST2) [79], hepatocyte growth factor [80], mid-regional pro-adrenomedullin [81], and von Willebrand factor [82] may be useful to improve risk stratification in patients with AL amyloidosis, both in conjunction or substitution to the existing Mayo staging systems but, apart GDF-15 and ST2, they remain in early stages of clinical investigation and merit further clinical evaluation. Among the “omics” science, the transcriptomics explore the complete set of RNA transcripts from DNA in a cell or tissue. The transcriptome includes ribosomal, messenger, transfer RNA, other non-coding RNA and micro RNA (miRNA). Analysis of circulating miRNAs in amyloidosis is in their beginning but appears to show promising results. Weng et al. reported that several miRNAs are dysregulated and expressed at increased levels in bone marrow plasma cell from AL patients and that miRNA-16 is reduced in patients responsive to anti-plasma cell chemotherapy, but not in patients who had persistent disease [83]. Fishov et al. found that ten distinct miRNAs were significantly differently expressed in bone marrow samples from newly diagnosed AL amyloidosis patients compared to MM patients, and that many other miRNAs differed significantly among AL, MM patients and healthy controls, suggesting that these different patterns of miRNAs expression among the three groups, could be used to better understand the pathogenesis of the disease and predict the risk of progression to AL amyloidosis among patients with known plasma cell dyscrasia [84]. Circulating miRNAs have been also explored in patients with ATTRv and ATTRwt cardiomyopathy. Derda et al. in an array profiling experiments found 10 candidates being deregulated in TTR amyloidosis. Among them, the miR-339-3p was found up-regulated only in ATTRwt, but not in patients with heart failure of other origin or with ATTRv, supporting miR-339-3p as a potential candidate biomarker for ATTRwt [85]. Vita et al., exploring the miRNAs expression profile in a group of patients with ATTRv and neurological phenotype compared to those of patients whit Charcot Marie Toot disease and healthy controls, found a profound dysregulation in the expression of several miRNAs with some up-regulated and other down-regulated as a function of different stages of amyloid polyneuropathy. In particular, the serum level of miR-150-5p has been shown to discriminate well the symptomatic stage 1–2 compared to the asymptomatic stage 0 [86]. Although the study of miRNAs in amyloidosis is promising, it should nevertheless be emphasized that a strong evidence on their role as a specific biomarkers for diagnosis, risk stratification and treatment guidance currently has yet to be validated.

## Conclusion

In AL amyloidosis, biomarkers such as FLCs, natriuretic peptides and troponins are the most extensively studied and validated; they have proved useful to risk stratification, to guide treatment choice and to monitor hematological and organ response. Unlike AL, the role of circulating biomarkers (natriuretic peptides, TTR, RPB4) in the diagnosis and management of TTR-CA is much less defined and validated. The availability of disease-modifying agents highlights the need for new markers capable of addressing correct clinical management and more accurate prognostic stratification. In particular, markers are welcome to define the minimum disease threshold to justify the starting new treatments and to exclude patients with degrees of infiltration so advanced that they do not come any benefit from therapy or to identify “responders” and “non-responders” to a specific disease-modifying agent. Currently, no single or groups of biomarkers appear to possess these properties. A multiple biomarkers’ approach probably could offer better possibilities in the management of these patients, above all in the management of response to therapy.
